# Malignant Mesothelioma

**DOI:** 10.3390/cancers13143447

**Published:** 2021-07-09

**Authors:** Daniel L. Pouliquen, Joanna Kopecka

**Affiliations:** 1 Université d’Angers, Inserm, CRCINA, F-44000 Nantes, France; 2 Department of Oncology, University of Turin, 10126 Turin, Italy

Malignant mesothelioma (MM) is a rare and aggressive cancer, related to chronic inflammation and oxidative stress caused mainly by exposure to asbestos. Although this mineral has been banned for decades in many countries, epidemiologists predict the MM epidemic will last past 2040, raising many concerns in public health given its late diagnosis, dismal prognosis, and lack of current efficient therapies. To deal with this situation, important breakthroughs have recently been made in the understanding of MM’s complex biology and the carcinogenic process of the different patterns of the disease. Examples of these include the development of new biomarkers and the deciphering of gene-environment interactions, molecular mechanisms of invasiveness, deregulated pathways, altered expression of miRNAs, DNA damage repair or metabolic profiles. From this recent research, MM’s aggressive and chemoresistant character appears linked to a polyclonal malignancy, and heterogeneity in molecular alterations. Given these improvements, new therapeutic strategies are being explored to solve the double challenge faced by clinicians. The first is to reduce tumor development and its wasting consequences as soon as possible, without resistance and with limited toxicity. The second is to stimulate recognition of tumor cells by induction of a specific immune response. 

In this Special Issue, 168 authors representing 71 affiliations from 13 countries over three continents have made 19 contributions, and it is a great privilege and pleasure for the editors to introduce this collective work which summarizes important insights in this field of research. As MM is mostly related to genetic and epigenetic alterations caused by prolonged exposure to asbestos fibers, in the search for a noninvasive prognosis test, Cugliari et al. showed the potential predictive value of DNA methylation changes in white blood cells as a MM survival biomarker [1], and investigated differences between MM cases and asbestos-exposed cancer-free controls [2]. Another important question concerns the discrimination between MM and benign proliferation of mesothelial cells (also frequently treated by surgery), for which Shresta et al. propose a genomic differential characterization [3]. Although asbestos has been banned in many countries, epidemiologists predict the MM epidemic will probably last over 2040 as new environmental risks, such as carbon nanotubes, are emerging, as reviewed by Barbarino et al. [4]. The diagnosis of MM could now benefit from the developments of ancillary tests, as reviewed by Dipper et al. [5]. Moretti et al. also analyzed tumor biopsy and liquid biopsies from a set of patients and demonstrated that most mutated DNA can be detected into pleural fluids [6].

Fortunately, the last few years have also been characterized by important breakthroughs in improving our understanding of MM’s complex biology and in identifying biomarkers which could help early diagnosis and prognosis of this cancer. To limit potential sources of bias in the identification of such biomarkers, Nader et al. conducted cross-species proteomic analyses on three different MM sources [7]. To understand the role of the genes that relate to this disease, Karunakaran et al. constructed a MM interactome and identified five repurposable drugs targeting the interactome proteins [8]. Since immune therapy emerged as a promising treatment alternative, Vogl et al. reviewed the role of inflammatory parameters [9], and Napoli et al. examined the contribution of the tumor immune microenvironment [10]. As redox-sensitive transcription factors regulate cellular antioxidant defense, Schiavello et al. presented their potential as predictive biomarkers [11]. 

The involvement of the immune system is crucial in MM development and progression, however, as MM consists of different histological subtypes varying in aggressiveness, an important understanding of the biological background of its escape mechanisms was provided by Brcic, Mathilakathu et al. [12]. With the development of immune checkpoint therapy, Kitajima et al. also demonstrated how hybrid imaging modalities could contribute to therapy response assessment and predict prognosis [13]. Among other treatment strategies, Lee et al. showed how minimally invasive surgery may help manage short-term outcomes of patients with MM [14], while Di Gregorio et al. revealed how metabolomic changes are associated with clinical outcomes following radical hemithoracic radiotherapy [15]. In the search for new targets, Yang et al. demonstrated that the exploration of aberrant biochemical networks and potential drug vulnerabilities induced by tumor suppressor loss provide interesting prospects for the treatment of MM [16]. Moreover, Anobile, Bironzo et al. evaluated the preclinical efficacy of a new marine-derived anticancer drug in patient-derived samples of MM [17]. Finally, Kotecha, Tonse et al. provided baseline comparative values in their review of survival of MM patients treated with systemic therapy combinations for locally, advanced, or metastatic disease [18], and Dulloo et al. reviewed new opportunities in molecular strategy therapy for this cancer [19]. 

Malignant mesothelioma still represents a devastating disease, and the final goal of all our research efforts is to provide prolonged survival with maintained quality of life to patients. The 19 articles contained in this Special Issue, which cover multiple and complementary aspects of this research, might contribute to reach this goal in the future, while opening interesting prospects for improving both the early diagnosis and treatment of this cancer. This collective work is also a good illustration of continued collaboration between disciplines and research teams all over the world, which could provide a basis for the emergence of new ideas and concepts in this field.
